# Supplementary figures and images for: EGFR-MEK1/2 cascade negatively regulates bactericidal function of bone marrow macrophages in mice with Staphylococcus aureus osteomyelitis
Source: PLoS Pathog. 2024 Aug 5;20(8):e1012437. doi: 10.1371/journal.ppat.1012437 (PMC11326603; doi:10.1371/journal.ppat.1012437)

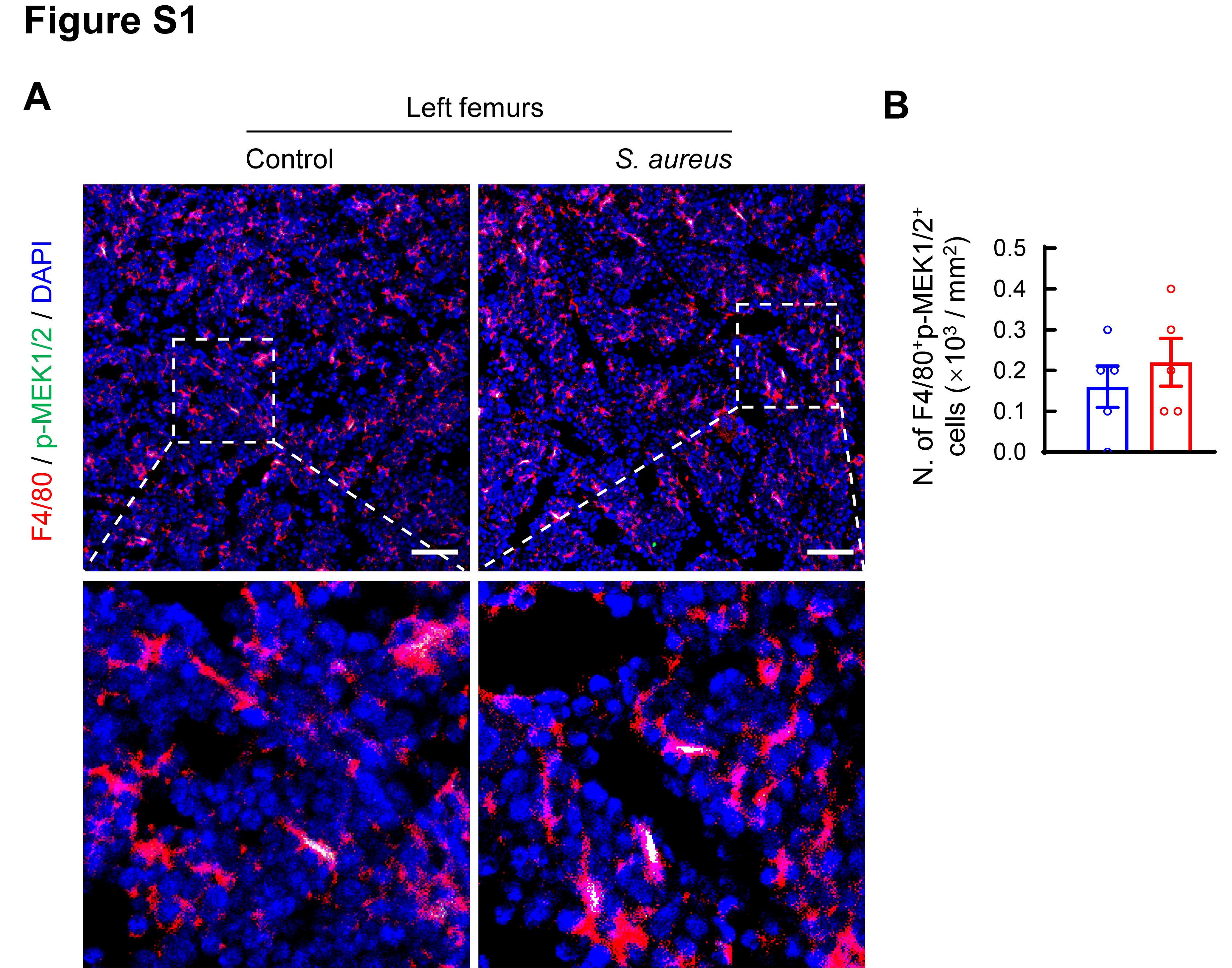

Supplement: S1 Fig — (A) Representative images of immunofluorescence staining for F4/80+ (red) and p-MEK1/2+ (green) cells in femoral bone marrow. (B) Quantification of the number of F4/80+p-MEK1/2+ cells in the field of view. Scale bars, 50 μm. n = 5/group, Student’s t test. (TIF) [file ppat.1012437.s001.tif]

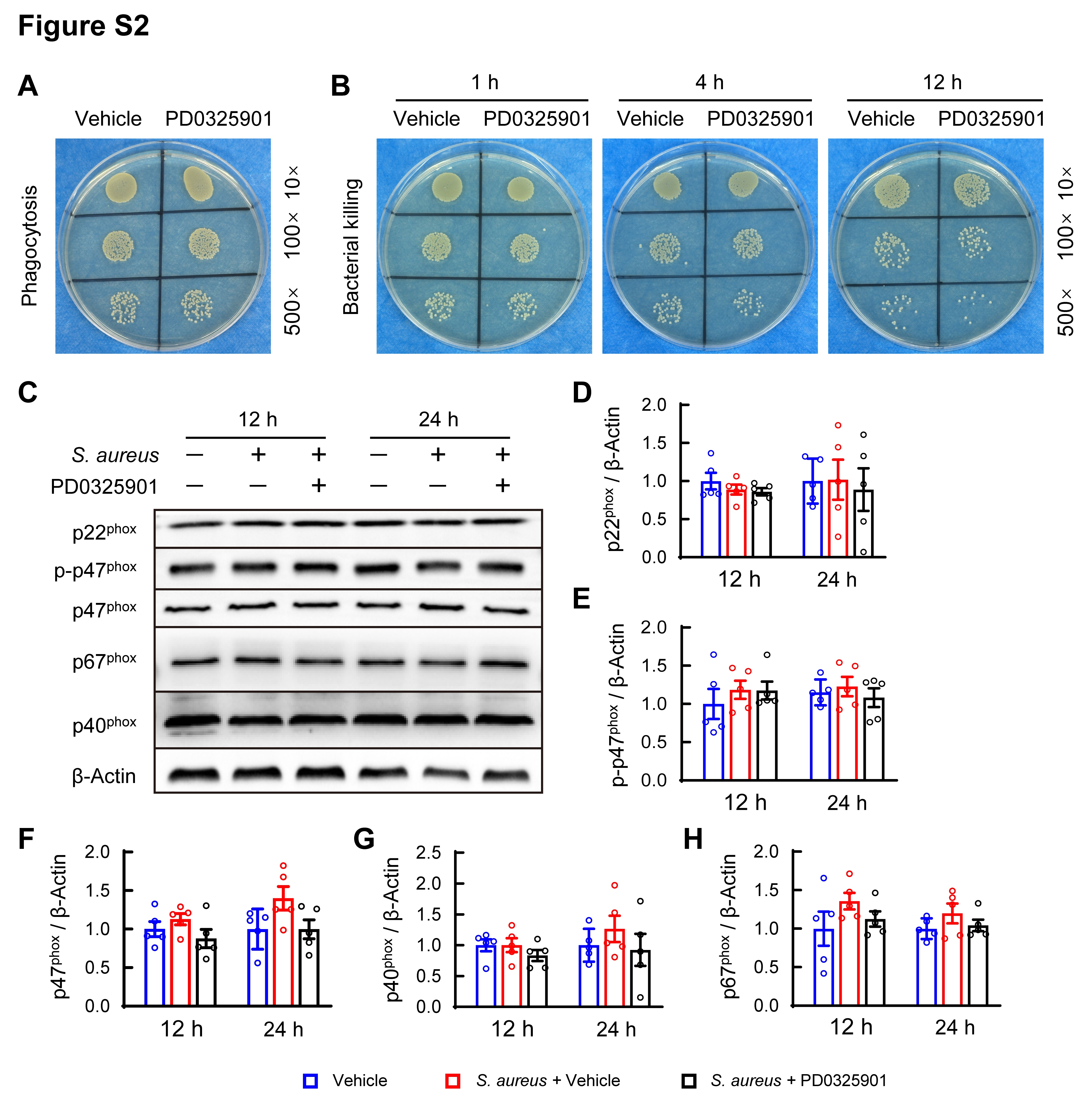

Supplement: S2 Fig — (A) Representative images of S. aureus colonies for phagocytosis assay. (B) Representative images of S. aureus colonies for the bactericidal assay. (C) Representative western blot images and (D) Quantification of relative levels of p22phox, p-p47phox, p47phox, p67phox, and p40phox. n = 5/group, one-way ANOVA with Tukey’s post-hoc test was used. (TIF) [file ppat.1012437.s002.tif]

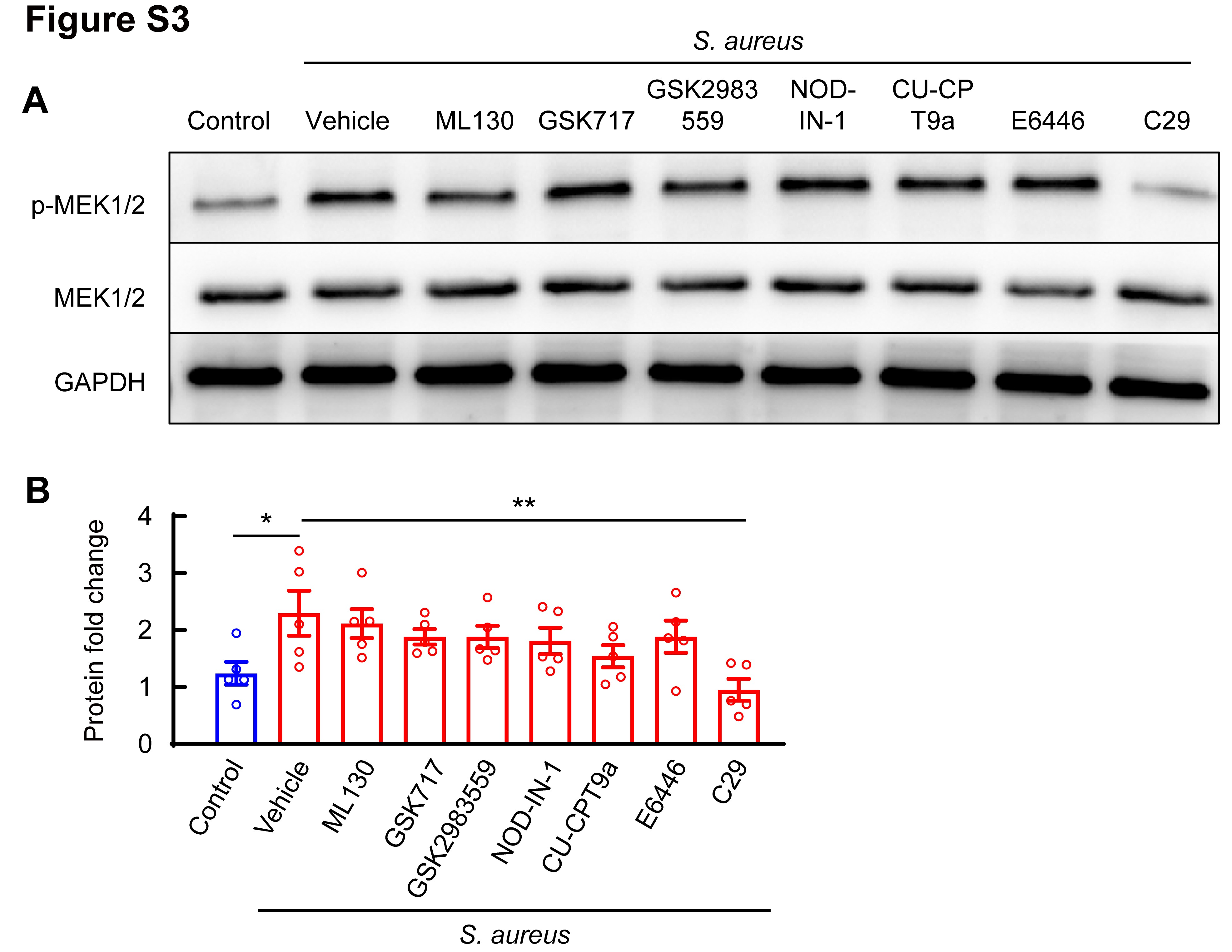

Supplement: S3 Fig — (A) Representative images and (B) Western blot quantification for p-MEK1/2 and MEK1/2. n = 5/group, *p < 0.05, **p < 0.01, one-way ANOVA with Dunnett’s post-hoc test was used. (TIF) [file ppat.1012437.s003.tif]

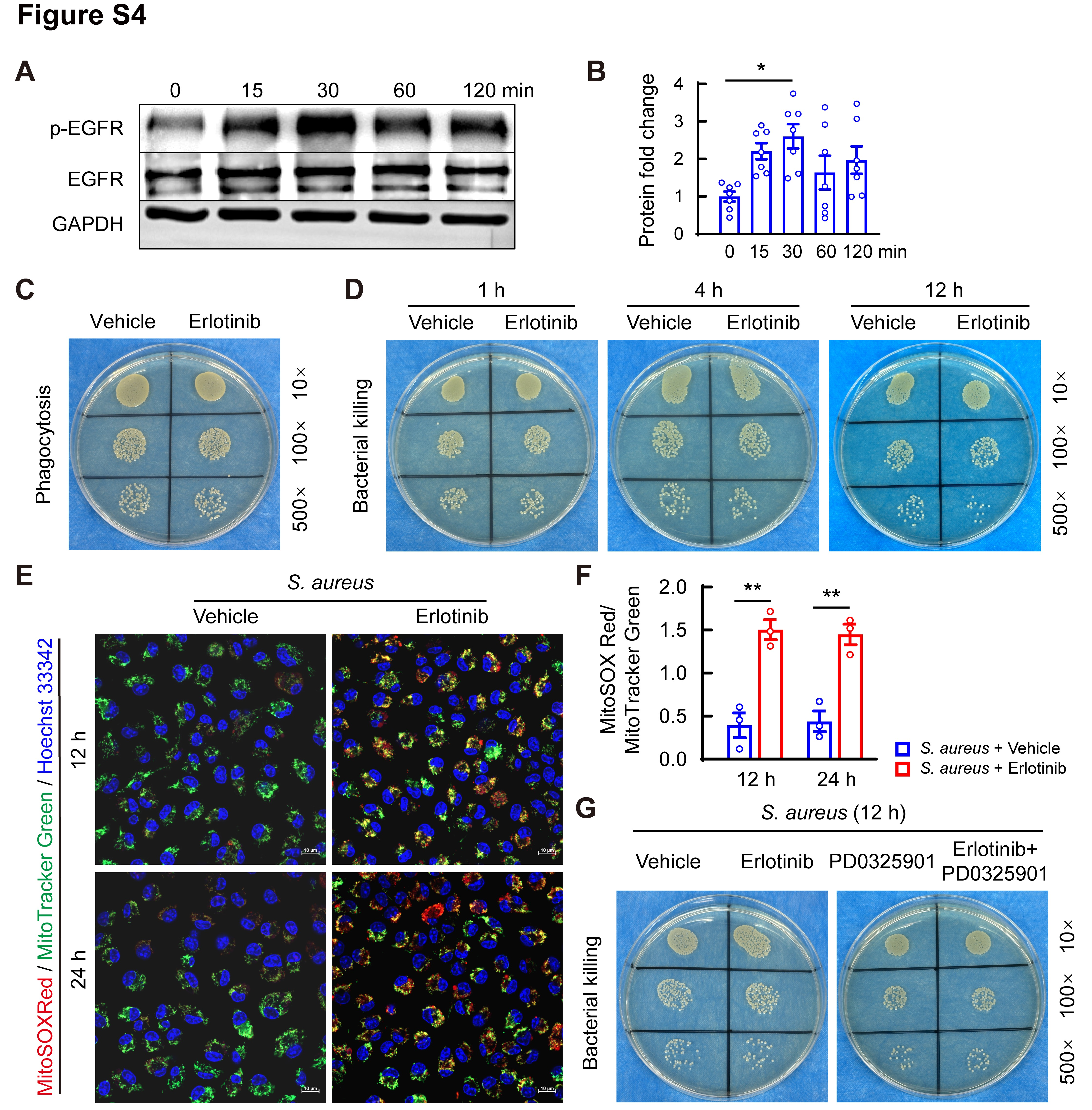

Supplement: S4 Fig — (A) Representative images of western blots and (B) Quantification of p-EGFR levels relative to total EGFR in BMDMs infected by S. aureus (MOI = 10) for the indicated time. n = 7/group. * p < 0.05 versus control, one-way ANOVA with Tukey’s post-hoc test used. BMDMs were pretreated with 10 μM erlotinib or vehicle (DMSO) for 1h, followed by S. aureus challenge for the indicated time. (C) Representative image of S. aureus colonies for phagocytosis assay. (D) Representative images of intracellular colonies of S. aureus for the bactericidal assay. BMDMs were infected with S. aureus at MOI of 10 for 1 h. After removing non-phagocytosed extracellular bacteria, cells were treated with 10 μM erlotinib or vehicle (DMSO) for the indicated time points. (E) Representative images and (F) Quantification of mtROS in BMDMs detected using MitoTracker Green and MitoSOX Red. Nuclei were stained with Hoechst 33342. Cells were infected with S. aureus (MOI = 10) for 1 h. After removing non-phagocytosed extracellular bacteria, cells were treated with 10 μM erlotinib or vehicle (DMSO) for 12 h and 24 h. Scale bars, 10 μm. n = 3/group. ** p < 0.01, Student’s t test was used. (G) Representative images of intracellular colonies of S. aureus for the bactericidal assay. (TIF) [file ppat.1012437.s004.tif]

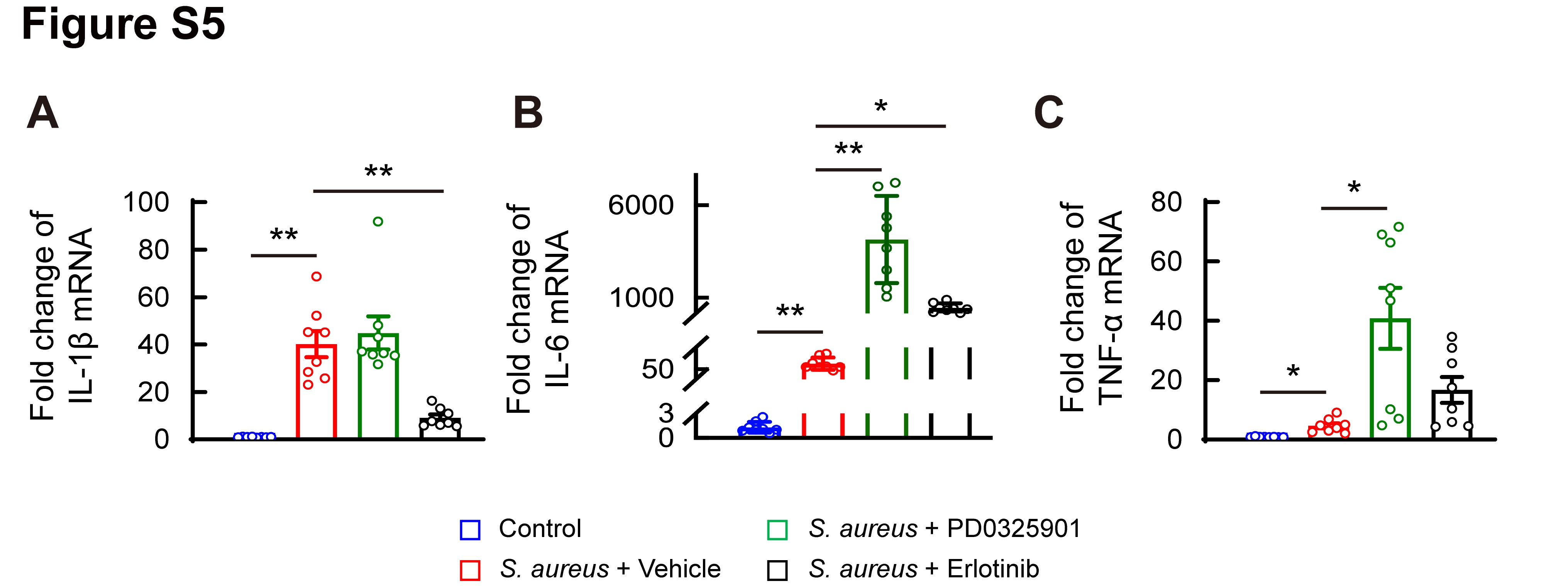

Supplement: S5 Fig — BMDMs were infected with S. aureus at MOI of 10 for 1 h. After removing non-phagocytosed extracellular bacteria, cells were treated with 10 μM erlotinib, 1 μM PD0325901, or vehicle for 12 h. n = 8/group, * p < 0.05, ** p < 0.01, one-way ANOVA with Dunnett’s post-hoc test used. (TIF) [file ppat.1012437.s005.tif]

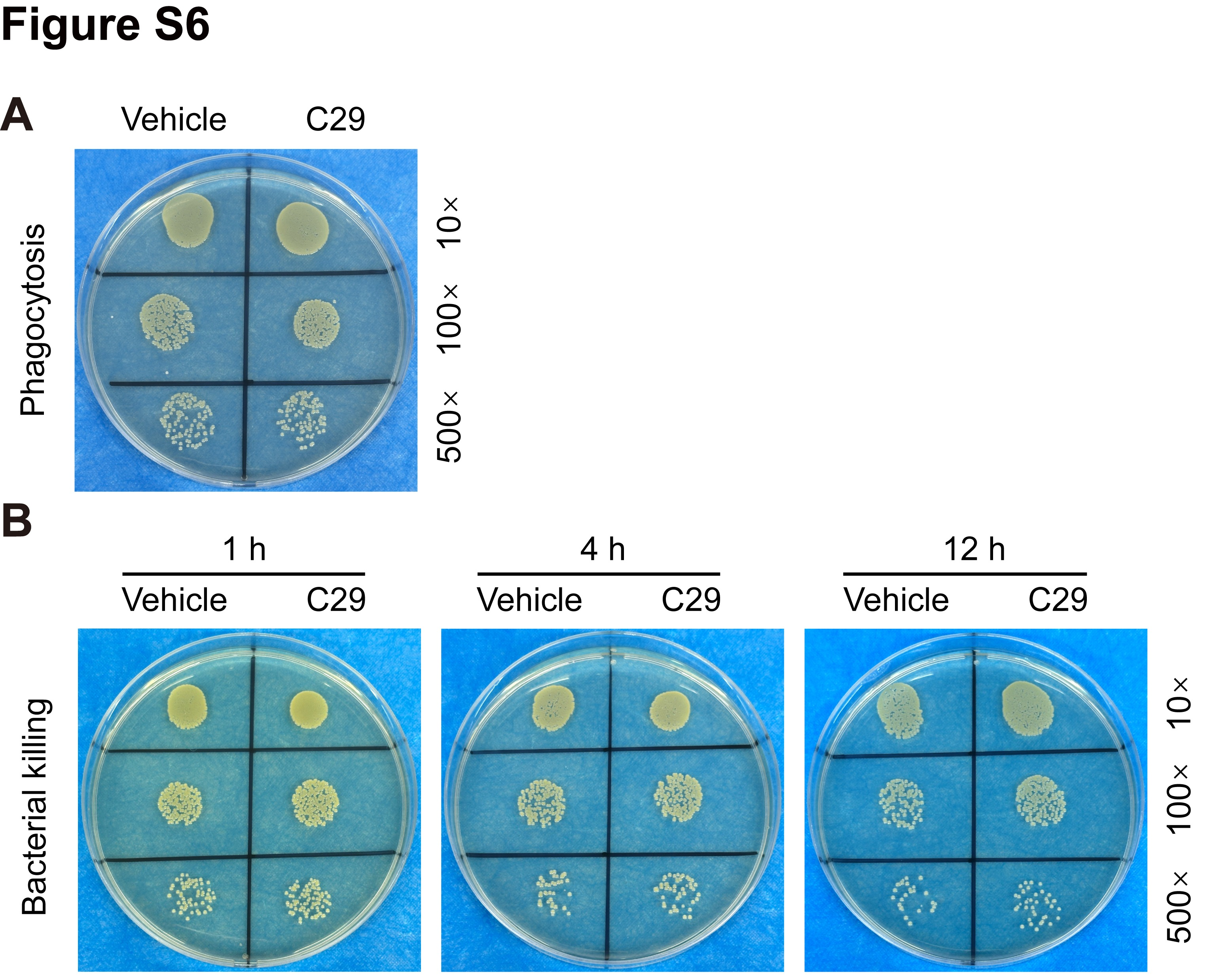

Supplement: S6 Fig — (A) Representative image of intracellular colonies of S. aureus for phagocytosis assay. (B) Representative images of intracellular colonies of S. aureus for bacterial killing assay. (TIF) [file ppat.1012437.s006.tif]

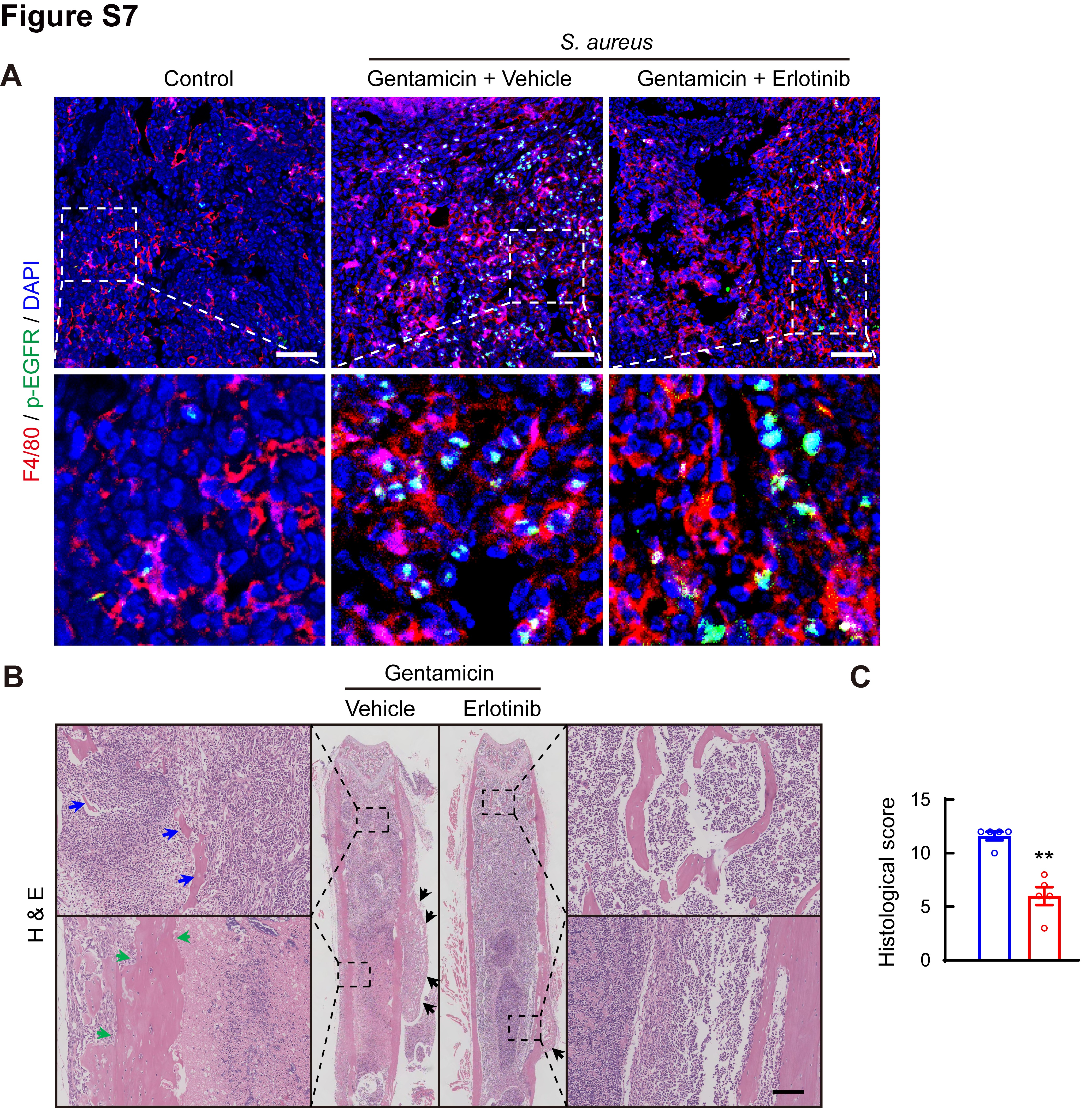

Supplement: S7 Fig — (A) Representative images of immunofluorescence staining for p-EGFR in F4/80+ macrophages in the right femoral bone marrow of S. aureus-infected mice treated with erlotinib or vehicle, and control mice without infection. (B) Representative images of H&E staining for S. aureus-infected femurs from mice treated with erlotinib and vehicle. (C) Quantification of histopathological changes using the scoring system established by Smeltzer et al [51]. Blue arrows show trabecular bone with empty lacunae, green arrows show erosion in cortical bone, and black arrows show reactive new bone formation around cortical bone. Scale bar, 100 μm. n = 5/group, **p < 0.05, Mann Whitney test. (TIF) [file ppat.1012437.s007.tif]
