# Supplementary material for: EGFR-MEK1/2 cascade negatively regulates bactericidal function of bone marrow macrophages in mice with Staphylococcus aureus osteomyelitis
Source: PLoS Pathog. 2024 Aug 5;20(8):e1012437. doi: 10.1371/journal.ppat.1012437 (PMC11326603; doi:10.1371/journal.ppat.1012437)
Supplement: Attachment — Submitted filename: Reviewer response PP.docx [file ppat.1012437.s010.docx]

Reviewer’s response

The manuscript written by Wu, X. et al. submitted to *Plos Pathogens* studies the role of the EGFR/MEK pathway in *S. aureus* killing by macrophages and conclude that the bacterium upregulates this signaling pathway to increase its survival within macrophages that likely contributes to its persistent infection seen in osteomyelitis. The authors established an *in vivo* model of *S. aureus* osteomyelitis in mice and showcase the beneficial *in vivo* effects of the MEK inhibitor, PD0325901. The report also investigates the role of EGFR, mitoROS and Chek2, a positive regulator for mtROS production. The data are interesting and represent a novel mechanism by which *S. aureus* seems to promote its persistence in host macrophages. However, the detailed signaling mechanism is largely based on in vitro experiments using BMDMs infected with S. aureus. Additional in vivo experiments focusing on key points of the signaling mechanisms revealed in vitro would further increase the impact of the work. Also, exploring phagosomal, not mitochondrial, ROS production is missing and is needed to understand the whole picture. Please find below my major and minor comments.

Major comments

- Line 112. If I am reading it correctly, the authors refer to the PD compound as an adjuvant to gentamicin. Vaccines are not used in this paper, so it remains unclear to me why the PD compound is referred to in the paper as an adjuvant?
- The authors should explain why was the use of gentamicin needed in the osteomyelitis model? Gentamicin was used to prevent a systemic S. aureus infection or infections due to potential contaminating microorganisms? Please add clarification about this.
- Data shown in Figures 3C and D were only done three times and show a wide spread. Therefore, more experiments should be performed to support the authors’ related claim that the PD inhibitor does not affect S. aureus phagocytosis by BMDMs.
- The authors should show that erlotinib reduces EGFR phosphorylation as they expect it
- While the investigators indicate the involvement of the EGFR/MEK/mROS and Chek2 signaling in S. aureus persistence in macrophages in vitro, most of these data were not proven in vivo, in their osteomyelitis model. Testing some of these inhibitors in their mouse model would be important to increase the clinical relevance of their findings. Alternatively, the authors could test their model in mice deficient in Tlr2 or Egfr.
- Mitochondrial ROS production has been implicated as important in microbial killing of phagocytes including macrophages, its exact mechanism of action remains largely unclear. The investigators should also comment and provide data on a much better accepted and better understood killing mechanism of *S. aureus*, phagocytic ROS production. It is known to be critical for *S. aureus* killing. Please look into phagosomal ROS production or phosphorylation of NADPH oxidase components in S. aureus -phagocytosing macrophages.

Minor comments

- The manuscript should be edited by a native English speaker
- Line 117-118. The T.b.. T.h. and P.f. abbreviations should be introduced at their first mentioning.
