# Supplementary material for: EGFR-MEK1/2 cascade negatively regulates bactericidal function of bone marrow macrophages in mice with Staphylococcus aureus osteomyelitis
Source: PLoS Pathog. 2024 Aug 5;20(8):e1012437. doi: 10.1371/journal.ppat.1012437 (PMC11326603; doi:10.1371/journal.ppat.1012437)
Supplement: Attachment — Submitted filename: Response to Reviewers.docx [file ppat.1012437.s011.docx]

**Part I - Summary**
Please use this section to discuss strengths/weaknesses of study, novelty/significance, general execution and scholarship.

**Reviewer #1:**

*In the submitted manuscript Wu et al demonstrate MEK1/2 putatively inhibits Staphylococcal killing by macrophages, and that blocking of this pathway enhances outcomes in an osteomyelitis model of infection. The manuscript is marked by impressive outcomes in the preclinical model that is used. However, small cohorts used for in vitro studies are problematic and strongly diminish the rigor of the conclusions made by the authors. These should be addressed*

**Response:** We agree with the reviewer that 3 replicates for *in vitro* studies were too small. We have repeated most *in vitro* experiments independently for 8 times. New data are summarized in Figures 3B-3D, 3F, 4C, 4F-4H, 4J, 5B-5E, 5G, 6F-6H, and 6J.

**Reviewer #2:**

*The manuscript “EGFR-MEK1/2 cascade negatively regulates bactericidal function of bone marrow macrophages in mice with Staphylococcus aureus osteomyelitis” by Wu et al. investigates the role of the MEK1/2 pathway in a Staphylococcus aureus osteomyelitis in a mouse infection model, along with transcriptomic analysis and in vitro methodology. They demonstrate that S. aureus osteomyelitis infection in mice activates the EGFR-MEK1/2 cascade resulting in a reduction in mtROS levels, dampening the bactericidal abilities of macrophages. This has interesting potential if blocking such pathways can be combined with antibiotic treatment in vivo, but further research is required to establish this. In general this is a thoroughly researched manuscript but in my opinion requires revisions before accepting for publication, including expanding upon how some conclusions have been made. A lot of data is shown but I suggest some of it can be moved to supplementary figures to make the main figures clearer to interpret. My comments to the authors are detailed below.*

**Response:** We appreciate the reviewer’s positive comments and constructive suggestions. We have evaluated the role of EGFR signaling and its interaction with MEK1/2 in regulating bactericidal function, mtROS production, and Chek2 expression in primary BMDMs. Additionally, we assessed the effect of pharmacological inhibition of EGFR signaling on the expression of MEK1/2-Chek2 axis, bacterial load in infected femurs, and alterations of bone microstructure in *S. aureus* osteomyelitis mouse model. Our *in vitro* and *in vivo* data indicate that overactivation of the EGFR-MEK1/2 pathway may reduce mtROS levels by suppressing Chek2 expression in macrophages, impairing macrophage bactericidal function, and promoting *S. aureus* osteomyelitis pathogenesis in mice.

Following the reviewer’s suggestion, we moved some data to supplementary figures, including images of agar plates for the phagocytosis/bacterial killing assays, negative western blot data for phagocyte NADPH oxidase components, and western blot data for time-dependent activation of EGFR signaling, et al.

The new data have been incorporated into the manuscript, as detailed in part II.

***Reviewer #3:***

*The manuscript written by Wu, X. et al. submitted to Plos Pathogens studies the role of the EGFR/MEK pathway in S. aureus killing by macrophages and conclude that the bacterium upregulates this signaling pathway to increase its survival within macrophages that likely contributes to its persistent infection seen in osteomyelitis. The authors established an in vivo model of S. aureus osteomyelitis in mice and showcase the beneficial in vivo effects of the MEK inhibitor, PD0325901. The report also investigates the role of EGFR, mitoROS and Chek2, a positive regulator for mtROS production. The data are interesting and represent a novel mechanism by which S. aureus seems to promote its persistence in host macrophages. However, the detailed signaling mechanism is largely based on in vitro experiments using BMDMs infected with S. aureus. Additional in vivo experiments focusing on key points of the signaling mechanisms revealed in vitro would further increase the impact of the work. Also, exploring phagosomal, not mitochondrial, ROS production is missing and is needed to understand the whole picture. Please find below my major and minor comments.*

**Response:** We appreciate the reviewer’s positive comments and helpful suggestions. As suggested by the reviewer, we investigated whether blocking EGFR signaling could reduce bacterial load and rescue bone destruction, and examined changes in p-MEK1/2 and Chek2 expression in F4/80+ macrophages in *S. aureus* osteomyelitis mouse model. In primary BMDMs, we evaluated expression levels of phagocyte NADPH oxidase components after persistent *S. aureus* infection and the effect of MEK1/2 pathway inhibition on their expression. The data are summarized the new figures detailed in Part II.

***Part II – Major Issues: Key Experiments Required for Acceptance*** *Please use this section to detail the key new experiments or modifications of existing experiments that should be absolutely required to validate study conclusions.
Generally, there should be no more than 3 such required experiments or major modifications for a "Major Revision" recommendation. If more than 3 experiments are necessary to validate the study conclusions, then you are encouraged to recommend "Reject".*

***Reviewer #1:***

***1.*** *The greatest issue I have for the paper is the paucity of replicates of the in vitro work. The authors have multiple figures, some which are critical to support the conclusions of the work, that have n=3-4 (e.g. Fig 3A, 3F, 4I, 4K…). These are in vitro experiments and the n= should be at least 8.*

**Response:** Thank the reviewer for pointing out this issue. We have repeated most *in vitro* experiments independently for 8 times. New data are summarized in Figures 3B-3D, 3F, 4C, 4F-4H, 4J, 5B-5E, 5G, 6F-6H, and 6J.

***2.*** *In addition killing rates in Fig 4 and Fig 5E, should be demonstrated in fashion similar to Fig 3F, and multiple time points are shown*

**Response**: We evaluated the effect of erlotinib and C29 on bactericidal function at multiple time points, as suggested by the reviewer. Our results showed that both erlotinib and C29 treatment had no significant effect on bactericidal function at earlier time points of infection (1 h and 4 h). After 12 h of infection, erlotinib notably enhanced the bacterial killing rate of the BMDMs (Figure 4G, Figure S4D), whereas C29 treatment decreased the bacterial killing rate and increased the bacterial burden in BMDMs (Figure 5E, Figure S6B). These new data have been incorporated into the manuscript. (Lines 210-211, 238-240)

***3.*** *Why is Erlotinib and 325901 not tested together? This is relevant.*

**Response**: We thank the reviewer for this constructive suggestion. We assessed the effect of a combination of erlotinib and PD0325901 on bacterial killing, mtROS production, and Chek2 expression in BMDMs, following the reviewer’s suggestion.

We evaluated whether combining erlotinib and PD0325901 could further enhance the bactericidal function of BMDMs after 12 h of infection. Unexpectedly, no synergistic effect was observed (Figure 4H, Figure S4G). MitoSOX staining showed that both PD0325901 and erlotinib treatment upregulated mtROS levels, but their combination did not further increase mtROS levels after 12 h of *S. aureus* infection (Figure 4I and 4J). Similarly, combined treatment with PD0325901 and erlotinib did not further increase Chek2 protein levels (Figure 6E-6H). New data and figures have been incorporated into the manuscript. (Lines 214-218, 260-262)

***Reviewer #2:***

***1.*** *Figure 2: In lines 144-146 you state “Our data clearly showed that the levels of p-MEK1/2 were upregulated predominantly in F4/80+ macrophages surrounding the abscess”. Did you investigate p-MEK1/2 levels in F4/80+ macrophages away from the abscess, for example in the left femur of the S. aureus infected mice, to support your statement that MEK1/2 levels are increased specifically at the abscess site? Please include this data if so, otherwise adjust your conclusions accordingly to e.g. “Our data clearly showed that the levels of p-MEK1/2 were upregulated in F4/80+ macrophages of S. aureus infected mice compared to uninfected controls”.*

**Response**: We thank the reviewer for this constructive comment. We have evaluated p-MEK1/2 levels in F4/80+ macrophages in both right femurs (surgery and infected side) and left femurs (contralateral side) of mice with *S. aureus* osteomyelitis and control ones. Specifically, we quantified the number of F4/80^+^p-MEK1/2^+^ cells in the area around the abscess and away from the abscess in the right femurs of *S. aureus*-infected mice and in left femoral bone marrow in the field of view were quantified.

Our data showed a significant increase in F4/80^+^p-MEK1/2^+^ cells in the bone marrow away from the abscess in the *S. aureus*-infected right femurs compared with control mice (Figure 2C and 2D). Notably, p-MEK1/2 levels were predominantly upregulated in F4/80^+^ macrophages surrounding the abscess (Figure 2C and 2D). The levels of F4/80^+^p-MEK1/2^+^ cells in the left femoral bone marrow (contralateral side of the surgery and infection) of *S. aureus*-infected mice were comparable to those in control mice (Figure S1). These data suggest that MEK1/2 pathway is primarily activated in F4/80^+^ macrophages surrounding the abscess. New data have been incorporated into the manuscript. (Lines 142-149, 771-776)

**Reviewer #3:**

***1.*** *The authors should show that erlotinib reduces EGFR phosphorylation as they expect it*

**Response**: We thank the reviewer for pointing out this issue. We evaluated the inhibitory effect of erlotinib on EGFR signaling *in vivo* using immunofluorescence staining. Compared with control mice without infection, there was extensive p-EGFR expression in F4/80^+^ macrophages in the bone marrow of *S. aureus*-infected femurs of mice treated with vehicle. As expected, p-EGFR level was considerably suppressed in *S. aureus*-infected femurs of mice treated with erlotinib (Figure S7A). This effect has been addressed in the manuscript. (Lines 271-273)

***2.*** *While the investigators indicate the involvement of the EGFR/MEK/mROS and Chek2 signaling in S. aureus persistence in macrophages in vitro, most of these data were not proven in vivo, in their osteomyelitis model. Testing some of these inhibitors in their mouse model would be important to increase the clinical relevance of their findings. Alternatively, the authors could test their model in mice deficient in Tlr2 or Egfr.*

**Response**: Thank the reviewer for this constructive suggestion. To assess the role of EGFR signaling in the pathogenesis of *S. aureus* osteomyelitis in mice, *S. aureus*-infected mice were randomly subjected to treatment with erlotinib (10 mg/kg/day, i.p., #HY-1208, MCE) or vehicle (10% DMSO in corn oil) from day 5 post-infection. All mice were treated with gentamicin (20 mg/kg/day, i.p.) from day 1 post-surgery to reduce extracellular *S. aureus* burden in the infected femurs. Mice were euthanized on day 14 after surgery, and their implanted right femurs were collected for further analysis.

We observed that erlotinib-treated mice had significantly reduced bacterial load in *S. aureus*-infected femurs compared with vehicle-treated mice (Figure 7A and 7B). Micro-CT imaging showed that erlotinib treatment significantly reduced reactive new bone formation around cortical bone and decreased cortical bone loss (Figure 7C-7E). Consistently, erlotinib-treated mice showed notably increased bone mineral density (BMD) and the ratio of bone volume to total volume (BV/TV), primarily due to rescued trabecular bone number (Tb.N) (Figure 7C, 7F-7H). However, there were no changes in trabecular bone thickness (Tb.Th) and trabecular pattern factor (Tb.Pf) in erlotinib-treated mice compared with vehicle-treated ones (Figure 7I and 7J). Histological staining and histopathological scoring analysis confirmed the positive effect of erlotinib treatment on bone structure in mice with *S. aureus* osteomyelitis (Figure S7B and S7C). Specifically, vehicle-treated mice showed deformed femurs with extensive abscess formation in the bone cavity and loss of trabecular bone in the metaphyseal area, whereas erlotinib-treated mice had improved trabecular bone structure with limited abscess formation around the implant (Figure S7A). These data indicate that blocking EGFR signaling alleviates bacterial burden and improves bone structure.

To confirm the role of EGFR signaling in the MEK1/2 pathway, we evaluated its expression in vehicle- and erlotinib-treated mice with *S. aureus* osteomyelitis. Immunofluorescence staining showed that erlotinib treatment substantially decreased p-MEK1/2 levels in F4/80^+^ cells (Figure 7K and 7L). We then evaluated whether inhibiting EGFR signaling could rescue Chek2 expression in macrophages. Results showed that erlotinib treatment markedly restored the decreased expression of Chek2 in *S. aureus*-infected femurs (Figure 7M and 7N). Together, our findings indicate that *S. aureus* infection may activate EGFR-MEK1/2 signaling, thereby suppressing mitochondrial ROS levels by downregulating Chek2 expression, leading to the progression of *S. aureus* osteomyelitis in mice.

These new data have been summarized in Figure 7 and incorporated into the manuscript. (Lines 273-297, 863-876)

***3.*** *Mitochondrial ROS production has been implicated as important in microbial killing of phagocytes including macrophages, its exact mechanism of action remains largely unclear. The investigators should also comment and provide data on a much better accepted and better understood killing mechanism of S. aureus, phagocytic ROS production. It is known to be critical for S. aureus killing. Please look into phagosomal ROS production or phosphorylation of NADPH oxidase components in S. aureus -phagocytosing macrophages.*

**Response**: We thank the reviewer for this constructive suggestion. Considering the crucial role of phagocyte NADPH oxidase in host defense by generating superoxide anion and other ROS molecules [24, 25], we evaluated the effect of PD0325901 on the total protein levels of NADPH oxidase components, including p22phox, NOX2/gp91phox, p47phox, p67phox, and p40phox. Surprisingly, the levels of these protein remained unchanged after 12 and 24 h of *S. aureus* infection (Figure S2C-S2H). However, PD0325901 treatment significantly upregulated NOX2 levels after 12 h and 24 h of *S. aureus* infection (Figure 3G and 3H). We inferred that the ability of BMDMs to produce ROS from phagocyte NADPH oxidase decreased to basal levels, insufficient for restricting *S. aureus* invasion after 12 h of persistent infection. Thus, overactivation of MEK1/2 may not only suppress mtROS production but also disrupt phagocytic ROS production in BMDMs under prolonged *S. aureus* infection. New data have been incorporated into the manuscript. (Lines 172-181, 793-795)

**Part III – Minor Issues: Editorial and Data Presentation Modifications**
Please use this section for editorial suggestions as well as relatively minor modifications of existing data that would enhance clarity.

**Reviewer #1:**

***1.*** *Line 79. Authors reference for S. aureus survival within macrophages as mechanism for chronic / relapsing infections. One reference, the review, is primarily focused on neutrophils. The other reference on the impact of PD-L1 signaling – neither paper specifically suggests that intracellular survival within macrophages is the mechanism for chronic infection. This statements should be modified, or references updated.*

**Response**: We updated the references to better match the statement regarding the role of intracellular persistence of *S. aureus* in providing a source for bacterial dissemination and promoting pathogenesis, as suggested by the reviewer. We have modified the statement in the manuscript accordingly. (Line 79)

Ref. 13. Jorch SK, Surewaard BG, Hossain M, Peiseler M, Deppermann C, Deng J, et al. Peritoneal GATA6+ macrophages function as a portal for *Staphylococcus aureus* dissemination. J Clin Invest. 2019; 129: 4643-4656.

Ref. 14. Tuffs SW, Goncheva MI, Xu SX, Craig HC, Kasper KJ, Choi J, et al. Superantigens promote *Staphylococcus aureus* bloodstream infection by eliciting pathogenic interferon-gamma production. Proc Natl Acad Sci USA. 2022, 119: e2115987119.

***2.*** *Line 113+. Lots of acronyms that are not described before. Their meaning can be found in the figure legend but should be in the text. Accordingly, Fig 1 is very complicated, and it is not clear what the readers should be looking at so as to gleam meaningful positive outcomes. This could be better described.*

**Response**: We have rearranged the data in the new Figure 1 and revised descriptions, providing full forms for all acronyms in the text, as suggested by the reviewer. (Lines 111-121)

***3.*** *Line 176. Why is TLR2 data not included as part of the findings presented here with the other TLRs? When reading this, I immediately asked myself what about TLR2 – why is it left out? Only to find it was examined later. The role of TLR2 helps to explain why EGFR inhibition is partial (line 188).*

**Response:** We appreciate the reviewer’s suggestions. Accordingly, we repeated the experiment to assess the effect of PRRs inhibitors, including the TLR2 inhibitor, on MEK1/2 pathway activation during S. aureus infection. New data has been added to the manuscript (Figure S3). (Lines 188-194)

**Reviewer #2:**

***1.*** *Supplementary figure 1: Please elaborate upon what the different treatments (5 μM ML130, 5 μM GSK717, 0.5 μM GSK2983, 10 μM NOD-IN-1, 5 μM CU-CPT9a, 1 μM E6446) target, explaining why they have been selected for this assay. Expand upon the statement in lines 176-177 “Results showed that neither TLR7/8/9…” to explain what the assay demonstrates. Please detail what the control is-is this vehicle treated too?*

**Response**: Thank the reviewer for pointing out these issues. To identify which PRR mediates MEK1/2 pathway activation in response to *S. aureus* infection, we used a series of specific inhibitors: ML130 for NOD1, GSK717 for NOD2, GSK2983559 for RIPK2, NOD-IN-1 for both NOD1 and NOD2, CU-CPT9a for TLR8, E6446 for TLR7 and TLR9, and C29 for TLR2. Our results showed that inhibition of TLR2 by C29 significantly blocked the activation of p-MEK1/2 by *S. aureus* infection, whereas TLR7/8/9 and NOD1/2 were not required for *S. aureus* to activate MEK1/2 pathway in macrophages (Figure S3A and S3B). We have added the above information to the “Results” section. (Lines 188-194)

To evaluate upstream signals involved in activating MEK1/2 pathway of BMDMs in response to *S. aureus* challenge, cells were pretreated with 5 μM ML130, 5 μM GSK717, 0.5 μM GSK2983559, 10 μM NOD-IN-1, 5 μM CU-CPT9a, 1 μM E6446, or the same volume of vehicle (DMSO, final dilution ratio: 1/1000) for 1 h, followed by *S. aureus* (MOI = 10) infection. Controls cells were treated with DMSO (1/1000) for 1h, followed by PBS (vehicle for *S. aureus* suspension, 7.5% in medium) for 30 min. We have provided the above information in the “Materials and Methods” section. (Lines 489-494)

***2.*** *Please detail what vehicle is and whether or not the same vehicle is used throughout all the assays in this manuscript.*

**Response**: Thank the reviewer for this helpful comment. To evaluate the effect of blocking MEK1/2 on the progression of *S. aureus* osteomyelitis in mice, *S. aureus*-infected mice were randomly assigned to treatment with PD0325901 (0.2 mg/kg/day, i.p., #S1036, Selleck) or vehicle (5% dimethyl sulfoxide (DMSO) in normal saline) from day 5 post-infection. To assess the role of EGFR signaling in the pathogenesis of *S. aureus* osteomyelitis in mice, *S. aureus*-infected mice were randomly subjected to treatment with erlotinib (10 mg/kg/day, i.p., #HY-1208, MCE) or vehicle (10% DMSO in corn oil) from day 5 post-infection.

For *in vitro* inhibitor treatment experiments, we used DMSO at a final dilution ratio of 1/1000 in culture medium.

We have added this vehicle information to the “Materials and Methods” section. (Lines 396, 399, 492-494, 519)

***3.*** *Line 95: Please change “improved bone destruction” to “rescued bone destruction”.*

**Response**: Thank the reviewer for this error, we have corrected it. (Line 94)

***4.*** *Figure 1 (and all subsequent figures): Please state what error bars indicate e.g. standard deviation, standard error of mean, and consistently show error bars in both directions.*

**Response**: Thank the reviewer for pointing out these issues. We have stated that all values were expressed as means ± standard error of mean (SEM) in the “Materials and Methods” section. (Line 565)

We have adjusted error bars in both directions as suggested.

***5.*** *Figure 1K (and throughout the rest of the figures): Please make the scale bars thicker and therefore easier to see.*

**Response**: We have made all scale bars thicker to make them easier to see, as suggested by the reviewer.

***6.*** *Figure 3G, 4L, 5F: Please state what the stain shown in blue is.*

**Response**: Thank the reviewer for pointing out this issue. We have labeled blue staining in all representative fluorescence images and stated them in figure legends.

***7.*** *Supplementary figure 2B: Please show the control group on the graph for example by adjusting the Y axis to make it visible.*

**Response**: We have adjusted the Y axis to make the control group visible on the graph, as suggested by the reviewer.

*8. Line 226: “Fig 6A and 5B” should read 6A and 6B.*

**Response**: We corrected a careless mistake, as noted by the reviewer. (Line 253)

***9.*** *Figure 6E: I do not think splitting the images into Chek2/DAPI and Chek2/MitoTracker Green adds anything to this figure, particularly as the DAPI staining is also shown in the Chek2/MitoTracker Green panel. I suggest removing the top panel and relabelling the remaining panel as Chek2/MitoTracker Green/DAPI.*

**Response**: We thank the reviewer for this constructive suggestion. Considering that MitoTracker is a probe for tracking mitochondria in live cells but not in fixed cell, we performed additional experiments to assess the subcellular localization of Chek2 with mitochondria labeled using antibody against TOM20. In the representative images, we labeled the panel as Chek2/Tom2/DAPI as suggested. New data have been summarized in Figures 6E-6H.

***10.*** *Line 254: Correct the spelling of “Salmaonella” to “Salmonella”.*

**Response**: Thank the reviewer for pointing out this error. We have corrected that spelling error. (Line 310)

***11.*** *Figures 3/4/5: I suggest the images of agar plates for the phagocytosis/bacterial killing assays could be made into supplementary figures instead, particularly as quantified data is shown in these figures too. This could allow for other panels of the figures to be made larger and overall the figures less crowded.*

**Response**: We have moved representative images of agar plates for the phagocytosis/bacterial killing assays into supplementary figures, as suggested by the reviewer.

***12.*** *Line 456: It states that Hoechst33324 was used to label nuclear DNA, but otherwise in the manuscript DAPI is specified. Please clarify which is the case and correct Hoechst33324 to 33342 if it was used.*

**Response**: We have corrected the error regarding Hoechst 33324, changing it to Hoechst 33342. For MitoSOX Red and MitoTracker Green staining, Hoechst 33342 was used to stain nucleus. For all other immunofluorescence staining, both in vitro and in vivo, DAPI was used. We have specified the reagent used for nuclear DNA staining in fluorescence images, the “Materials and Methods” section, and the Figure legends.

***Reviewer #3***

*Minor comments*

***1.*** *The manuscript should be edited by a native English speaker*

**Response**: We have carefully and rigorously reviewed and revised the manuscript. Additionally, we sought assistance from an expert in English to correct grammatical and formatting errors.

***2.*** *Line 117-118. The T.b.. T.h. and P.f. abbreviations should be introduced at their first mentioning.*

**Response**: We have provided the full form of those abbreviations in the manuscript, as suggested by the reviewer. (Lines 111-116)

***3.*** *Line 112. If I am reading it correctly, the authors refer to the PD compound as an adjuvant to gentamicin. Vaccines are not used in this paper, so it remains unclear to me why the PD compound is referred to in the paper as an adjuvant?*

**Response**: Thank the reviewer for pointing out this error. We refer to a combination treatment of the PD compound with gentamicin. We have revised it in the manuscript. (line 108)

***4.*** *The authors should explain why was the use of gentamicin needed in the osteomyelitis model? Gentamicin was used to prevent a systemic S. aureus infection or infections due to potential contaminating microorganisms? Please add clarification about this.*

**Response**: It was reported that gentamicin at 10 mg/kg had a poor killing effect on intracellular *S. aureus* in a mouse model of peritonitis [50], and our recent work showed a substantial amount of *S. aureus* resides in *Lyz2^+^* macrophages in the bone marrow of osteomyelitis mice treated with gentamicin at dose of 20 mg/kg/d [51]. To investigate the role and mechanism of intracellular *S. aureus* in pathogenesis of osteomyelitis, all mice were treated with gentamicin (20 mg/kg/day, i.p.) from day 1 post-surgery to reduce extracellular *S. aureus* burden in the infected femurs. We have addressed this point in manuscript. (Lines 403-408)

***5.*** *Data shown in Figures 3C and D were only done three times and show a wide spread. Therefore, more experiments should be performed to support the authors’ related claim that the PD inhibitor does not affect S. aureus phagocytosis by BMDMs.*

**Response**: Thank the reviewer for pointing out this issue, which is the same as the first comment raised by Reviewer #1 in Part II. We have repeated the *in vitro* experiments an additional 5 times using primary BMDMs isolated from different mice. Data have been added to new Figures 3B-3D, 3F, 4C, 4F-4H, 4J, 5B-5E, 5G, 6F-6H, and 6J.
