# Supplementary material for: EGFR-MEK1/2 cascade negatively regulates bactericidal function of bone marrow macrophages in mice with Staphylococcus aureus osteomyelitis
Source: PLoS Pathog. 2024 Aug 5;20(8):e1012437. doi: 10.1371/journal.ppat.1012437 (PMC11326603; doi:10.1371/journal.ppat.1012437)
Supplement: Attachment — Submitted filename: Response to Reviewers for 2nd revision.docx [file ppat.1012437.s012.docx]

**Responses to the comments of reviewers**

*1. The authors have appropriately responded to the reviewers criticisms with new data, increased numbers of replicates and clarification of the involvement of EGFR and TLR2 pathways. The grammar and syntax are also much improved however with remaining errors in the following lines that should be corrected:
l. 82, l161, l165, 1. 269 (should read "mice with implant associated osteomyelitis) - as well as many others remaining - usually in the lack of articles such as "a" or "the". Otherwise the manuscript is a nice contribution that very thoroughly details the role of this MEK/EGFR pathway in macrophage killing of S. aureus in a clinically relevant model.*

**Response**: We are grateful to the reviewers for their positive feedback and for highlighting the grammatical errors. We have corrected the errors in lines 82, 161, 165, and 269. Additionally, we thoroughly reviewed the manuscript and addressed any remaining issues, which are indicated using red-colored text.

*2. Important additional instructions*

*Figure Files:*

**Response**: All figures have been uploaded to the PACE digital diagnostic tool and adjusted to meet PLOS requirements.

*Data Requirements:*

**Response**: The source data used to generate the graphs has been uploaded as supporting information (lines 934-935). The transcriptome data has been deposited in the Gene Expression Omnibus (GEO) under accession number GSE272198 (lines 592-593).

*References:*

**Response**: We have verified all references to ensure they are complete and correct.
