# Supplementary material for: EGFR-MEK1/2 cascade negatively regulates bactericidal function of bone marrow macrophages in mice with Staphylococcus aureus osteomyelitis
Source: PLoS Pathog. 2024 Aug 5;20(8):e1012437. doi: 10.1371/journal.ppat.1012437 (PMC11326603; doi:10.1371/journal.ppat.1012437)
Supplement: Attachment — Submitted filename: Reviewer comments.pdf [file ppat.1012437.s009.pdf]

The manuscript “EGFR-MEK1/2 cascade negatively regulates bactericidal function of bone marrow macrophages in mice with Staphylococcus aureus osteomyelitis” by Wu *et al.* investigates the role of the MEK1/2 pathway in a *Staphylococcus aureus* osteomyelitis in a mouse infection model, along with transcriptomic analysis and *in vitro* methodology. They demonstrate that *S. aureus* osteomyelitis infection in mice activates the EGFR-MEK1/2 cascade resulting in a reduction in mtROS levels, dampening the bactericidal abilities of macrophages. This has interesting potential if blocking such pathways can be combined with antibiotic treatment *in* vivo, but further research is required to establish this. In general this is a thoroughly researched manuscript but in my opinion requires revisions before accepting for publication, including expanding upon how some conclusions have been made. A lot of data is shown but I suggest some of it can be moved to supplementary figures to make the main figures clearer to interpret. My comments to the authors are detailed below.

Major revisions:

Figure 2: In lines 144-146 you state “Our data clearly showed that the levels of p-MEK1/2 were upregulated predominantly in F4/80$^+$ macrophages surrounding the abscess”. Did you investigate p-MEK1/2 levels in F4/80$^+$ macrophages away from the abscess, for example in the left femur of the *S. aureus* infected mice, to support your statement that MEK1/2 levels are increased specifically at the abscess site? Please include this data if so, otherwise adjust your conclusions accordingly to e.g. “Our data clearly showed that the levels of p-MEK1/2 were upregulated in F4/80$^+$ macrophages of *S. aureus* infected mice compared to uninfected controls”.

Minor revisions:

Supplementary figure 1: Please elaborate upon what the different treatments (5 µM ML130, 5 µM GSK717, 0.5 µM GSK2983, 10 µM NOD-IN-1, 5 µM CU-CPT9a, 1 µM E6446) target, explaining why they have been selected for this assay. Expand upon the statement in lines 176-177 “Results showed that neither TLR7/8/9…” to explain what the assay demonstrates. Please detail what the control is – is this vehicle treated too?

Please detail what the vehicle is and whether or not the same vehicle is used throughout all the assays in this manuscript.

Line 95: Please change “improved bone destruction” to “rescued bone destruction”.

Figure 1 (and all subsequent figures): Please state what error bars indicate e.g. standard deviation, standard error of mean, and consistently show error bars in both directions.

Figure 1K (and throughout the rest of the figures): Please make the scale bars thicker and therefore easier to see.

Figure 3G, 4L, 5F: Please state what the stain shown in blue is.

Supplementary figure 2B: Please show the control group on the graph for example by adjusting the Y axis to make it visible.

Line 226: “Fig 6A and 5B” should read 6A and 6B.

Figure 6E: I do not think splitting the images into Chek2/DAPI and Chek2/MitoTracker Green adds anything to this figure, particularly as the DAPI staining is also shown in the Chek2/MitoTracker Green panel. I suggest removing the top panel and relabelling the remaining panel as Chek2/MitoTracker Green/DAPI.

Line 254: Correct the spelling of “*Salmaonella”* to “*Salmonella”*.

Figures 3/4/5: I suggest the images of agar plates for the phagocytosis/bacterial killing assays could be made into supplementary figures instead, particularly as quantified data is shown in these figures too. This could allow for other panels of the figures to be made larger and overall the figures less crowded.

Line 456: It states that Hoechst33324 was used to label nuclear DNA, but otherwise in the manuscript DAPI is specified. Please clarify which is the case and correct Hoechst33324 to 33342 if it was used.
